# Combination Therapy Using Inhalable GapmeR and Recombinant ACE2 for COVID-19

**DOI:** 10.3389/fmolb.2020.00197

**Published:** 2020-08-07

**Authors:** Navin Kumar Verma, Mobashar Hussain Urf Turabe Fazil, Shane P. Duggan, Dermot Kelleher

**Affiliations:** ^1^Lee Kong Chian School of Medicine, Nanyang Technological University, Singapore, Singapore; ^2^Faculty of Medicine, University of British Columbia, Vancouver, BC, Canada

**Keywords:** COVID-19 (2019-nCoV), GapmeR, angiotensin-converting enzyme 2, nasal delivery, therapy

## Abstract

Here we report our perspective on applying GapmeR technology in combination with recombinant angiotensin-converting enzyme 2 (ACE2) in the treatment of COVID-19 patients. GapmeR is a cell-permeating antisense single-stranded DNA molecule that can be designed to specifically target intracellular severe acute respiratory syndrome coronavirus 2 (SARS-CoV-2). Once internalized into host cells, such as lung alveolar cells, GapmeR molecules can bind to the viral RNA. This RNA/DNA hybrid will then be degraded by the RNase H enzyme abundantly present in the host cells. GapmeRs can be delivered to COVID-19 patients through inhalation or *via* nebulization. SARS-CoV-2-targeted GapmeR can also be given to frontline healthcare workers as a prophylactic protection. The recombinant ACE2 protein, the efficacy of which is being evaluated in clinical trials, will bind to the spike (S) glycoprotein of extracellular SARS-CoV-2 and potentially block viral infectivity. We propose that combining inhalable SARS-CoV-2-targeted GapmeRs with recombinant ACE2 could provide a viable and rapidly implementable more effective therapeutic approach for eradicating SARS-CoV-2 and save millions of lives.

## Introduction

COVID-19 or the severe acute respiratory syndrome coronavirus 2 (SARS-CoV-2) continues to threaten human health worldwide. As of 15th July 2020, there are over 13.4 million confirmed cases and over 580,000 deaths worldwide^1^. In an accelerated effort to develop therapeutic modalities and vaccines to manage this devastating pandemic, collaborative teams of regulatory bodies, health professionals, clinicians, and scientists in both academic and industrial settings have been engaged and integrated. Although there has been some success in developing new vaccines, repurposing previous coronavirus antibodies, and other potential therapeutic modalities, there is no effective therapy available for the ongoing COVID-19 pandemic. Through expedited regulatory processes, the US Food and Drug Administration (FDA) has granted Emergency Use Authorization (EUA) for remdesivir (an investigational drug, originally developed to tackle Ebola) on 1st May 2020 to treat severe COVID-19 cases (Media). Surprisingly, there has been relatively little exploration of directly targeting the SARS-CoV-2 genome.

## SARS-CoV-2

We now know the complete genome of SARS-CoV-2, which is a single-stranded positive-sense RNA of about ∼30 kb and is encapsulated within a membranous envelope. As with other coronavirus, SARS-CoV-2 contains four main structural proteins – spike (S) glycoprotein, membrane (M) protein, envelope (E) protein, and nucleocapsid (N) protein (Wu et al., 2020). The infection is established by virus particles following their entry through airway and binding of viral S protein to the receptor angiotensin-converting enzyme 2 (ACE2) expressed on type II alveolar cells of lung (Zou et al., 2020). The S-ACE2 complex then internalizes into alveolar cells by endocytosis (Yan et al., 2020). The ACE2 protein is also expressed on esophagus epithelial cells, cardiac muscle cells, ocular cells, kidney proximal tubules, urothelial cells, colon, and on the mucosa of oral cavity (Ma et al., 2020; Xu et al., 2020).

## Silencing the Viral Genome

RNA interference (RNAi) is a powerful biological process inherently applied by the host cells to destroy intracellular RNA viruses. For the past two decades, researchers have been widely capitalizing on RNAi mechanisms to silence cellular gene expression *in vitro* and *in vivo*. Studies have validated the use of small interfering RNA (siRNA) to safeguard host from viral infection by inhibiting the replication of viral genome, by blocking the expression of viral antigens and accessory genes and by interfering with the assembly of viral particles (Lenartowicz et al., 2016; Levanova and Poranen, 2018). Previous studies have demonstrated successful intratracheal administration of siRNA using pressurized metered dose inhalers or nebulizers (Miwata et al., 2018; Ng et al., 2019). Indeed, OliX Pharmaceuticals, Sirnaomics, and a collaborative team of Alnylam Pharmaceuticals and Vir Biotechnology have initiated the development of siRNA therapeutics to treat COVID-19 patients. However, siRNA-based therapeutic approach is challenging mainly because of poor stability of siRNA molecules in biological environment and problems associated with delivery into patients’ cells and tissues.

## Inhalable GapmeR as a Therapeutic Option for COVID-19

At this juncture, we propose to explore the utility of an alternative and possibly more effective approach using inhalable antisense DNA molecules, such as GapmeRs. Typically, GapmeR molecules have a central stretch of chemically modified DNA “gap” flanked by locked nucleic acids (LNA), which increase the binding affinity of GapmeR to the target RNA. In addition to specificity and highly efficient gene silencing, we have shown that GapmeRs can easily internalize into target cells (e.g., human primary and cultured T-cells) by macropinocytosis or gymnosis (Fazil et al., 2016; Verma et al., 2017; Ong et al., 2018); thus requiring no additional delivery assistance or transfection agents. GapmeR molecules targeting specific regions of SARS-CoV-2 RNA can easily be designed using publicly available genome sequence information and *in silico* antisense design tools (Kurreck et al., 2002). Care must be taken to avoid potential off-target effects by searching selected sequences of GapmeRs against the databases of human transcripts and filtering out cross-reacting sequences. Several other stringent parameters, including target accessibility, binding affinity and a series of assessment criteria as recommended by the Oligonucleotide Safety Working Group (Marlowe et al., 2017; Yoshida et al., 2019) should be taken into consideration.

**FIGURE 1:**
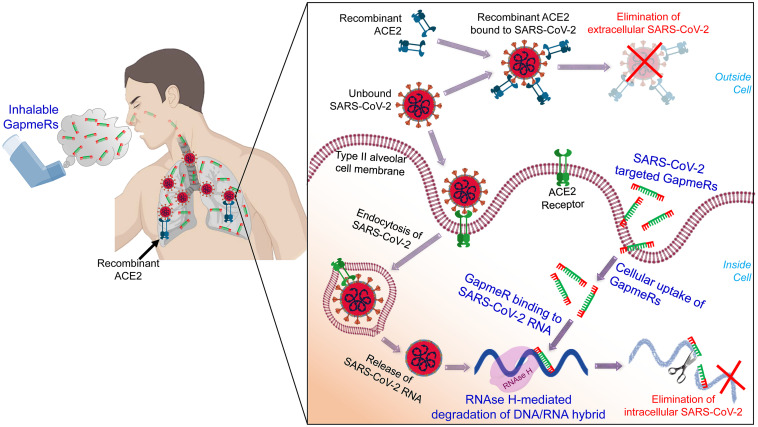
A schematic illustration of administering inhalable GapmeRs and recombinant ACE2 to the lung for treating COVID-19. The proposed mechanism of action of SARS-CoV-2 targeted GapmeR molecules in destroying intracellular viral RNA in type II alveolar cells and recombinant ACE2 in eliminating extracellular virus particles in the lung. Figure prepared using images created with BioRender.

We have already designed a number of GapmeR molecules targeting the highly conserved regions of the SARS-CoV-2, including the RNA-dependent RNA polymerase (RdRP), S protein and M protein. While designing SARS-CoV-2 targeting GapmeRs, we have considered multiple parameters, including RNA secondary structure, accessibility of target region, and avoidance of off-target effects using robust bioinformatics algorithms and *in silico* analysis. We believe that once internalized, virus-specific GapmeRs would bind to SARS-CoV-2 RNA within a host cell. RNase H enzymes present in the host cell will then specifically degrade the DNA/RNA hybrid and thus destroy SARS-CoV-2 (Figure 1).

GapmeRs can be administered *via* inhalation as aerosol or in powder form, either using a customized handheld nebulizer device or through the ventilator. Using either of these simple non-invasive inhalation devices, one can easily achieve high enough concentration of GapmeR molecules in the lung sufficient for type II alveolar cell uptake. GapmeRs could conceivably be administered to frontline healthcare worker dealing with COVID-19 patients to provide them prophylactic protection. Since GapmeR molecules are highly stable in human serum (Grünweller et al., 2003), we would expect less frequent dosing of the SARS-CoV-2-targeted GapmeRs, in this situation. GapmeRs may also remain effective or could be modified as the virus mutates and could have cross-reactivity against related viral strains that may emerge in the future. Other advantages of using GapmeR therapeutics are well-established chemistry and synthesis process, easily achievable manufacturing and bulk production and low storage or shipment cost due to high stability, even at room temperature.

It is now evident that GapmeR technology is gaining significant interest in therapeutic development (Hagedorn et al., 2018; Pedersen et al., 2020). There has been substantial progress since the first systemically delivered GapmeR therapeutic mipomersen (Kynamro) approved by the FDA in 2013 for reducing cholesterol levels in familial hypercholesterolemia (Parham and Goldberg, 2019). Tominersen (previously known as IONIS-HTTRx) is another GapmeR molecule that has been granted Orphan Drug Designation by FDA in 2016 for the treatment of Huntington’s disease (Rodrigues and Wild, 2018). Nusinersen (Spinraza), an antisense nucleotide with phosphorothioate backbones and 2′-O-methoxyethyl modification, has been approved by the FDA in 2016 for treating spinal muscular atrophy (Aartsma-Rus, 2017). More recently, in December 2019, FDA has granted accelerated approval to an antisense phosphorodiamidate morpholino oligomer, golodirsen, for treating patients with Duchene Muscular Dystrophy (Heo, 2020). GapmeR targeting SOD1 mutation, tofersen (previously known as IONIS SOD1Rx), is currently in phase III clinical trial (NCT02623699) for treating a fatal neurodegenerative disease amyotrophic lateral sclerosis. The continued success of GapmeR therapeutics and tolerable safety profiles in ongoing clinical trials predict a robust outlook for a new effective treatment strategy for COVID-19.

It is worth noting that, like any other drugs, GapmeRs may have unforeseen off-target effects, including for example, unwanted interactions with host proteins (e.g., Toll-Like Receptors). While such interactions between SARS-CoV-2-targeted GapmeRs and host proteins may not be strong, they may trigger unintended signaling pathways, cytokine release, and yield confounding physiological effects. In our previous work using gene expression analysis, we did not find any major immunological off-target effects of a panel of GapmeR molecules that we designed against multiple targets (Fazil et al., 2016). Nevertheless, if toxicity arises in further advanced studies, measures to eliminate or mitigate potential risks could be implemented during pre-clinical testing. Another challenge would be to efficiently deliver GapmeR molecules to type II alveolar cells in the lung. An antisense oligonucleotide eluforsen has been successfully administered *via* inhalation to treat respiratory symptoms in certain patients with cystic fibrosis and granted fast track designation by the FDA in 2016 (Kim et al., 2019). Single and multiple doses of inhaled eluforsen have been proven to be safe and well-tolerated (Drevinek et al., 2020). It would also be important to adjust the administering frequency and dosing of inhalable GapmeRs to manage varying levels of viremia as well as to treat patients at different stages of symptomatic or non-symptomatic infections. The key to success of translating GapmeR technology to treat COVID-19 patients would be to (*i*) screen multiple GapmeR designs against highly conserved regions of SARS-CoV-2 genome, (*ii*) carefully select potent candidates with high stability and no off-target effect, (*iii*) demonstrate their delivery to the lung using multimodal *in vitro*, organoids, and animal model systems appropriate for COVID-19 preclinical research (Takayama, 2020), (*iv*) determine dosing regimens for various infection scenarios, including for example, cases where the virus titer is increasing, and (*v*) achieve a good therapeutic window with acceptable toxicity profiles while suppressing viral loads in well-controlled experiments.

## Possibility of Combining SARS-CoV-2-Targeted GapmeR With Recombinant ACE2

In a recent study, Monteil et al. (2020) have shown the therapeutic potential of human recombinant soluble ACE2 (hrACE2) protein to inhibit the replication of SARS-CoV-2. In addition, hrACE2 protein has been shown to be protective in acute lung injury (Imai et al., 2005). Thus, soluble form of ACE2 would not only block the early entry of SARS-CoV-2 in host cells and hence viral spread, it would also protect the COVID-19 patients form severe acute lung failure (Zhang et al., 2020). Previous phase II trial results confirm that the hrACE2 is well-tolerated (even at high dose up to 0.8 mg/kg) in patients with acute respiratory distress syndrome (Khan et al., 2017) and significantly improves cardiac output and pulmonary vascular resistance in pulmonary arterial hypertension (Hemnes et al., 2018). Treatment of diabetic mice with hrACE2 (intraperitoneal injection of 2 mg/kg hrACE2 for 4 weeks) has been shown to slow the progression of diabetic nephropathy (Oudit et al., 2010). Accordingly, a number of clinical trials, including NCT04375046, NCT04335136, and NCT04382950, to evaluate the recombinant ACE2 therapy for COVID-19 have recently been initiated^2^. While viral replication is reduced through the use of hrsACE2, SARS-CoV-2 is not eliminated. It should be noted that SARS-CoV-2 can target multiple cell types expressing ACE2 and that, in addition to alveolar epithelial cells, many human tissues and organs express the ACE2 (Xu et al., 2020; Yan et al., 2020; Zou et al., 2020), which might provide possible routes of entry for the SARS-CoV-2. Anticipating successful attenuation of outcomes in these clinical trials for recombinant ACE2, we propose that simultaneously administration SARS-CoV-2-targeted GapmeR through inhalation route could be synergistic in fully inhibiting viral replication. While recombinant ACE2 would block the extracellular virus particles entering host cells, therapeutic GapmeR would destroy intracellular SARS-CoV-2 genome (Figure 1). This combinatorial approach could thus eradicate SARS-CoV-2, effectively reduce the severity of infection and save millions of lives.

## Conclusion

In conclusion, there is an urgent need to radically rethink current management of the devastating COVID-19 pandemic. Although there are a set of rapidly increasing challenges, logistic, and regulatory hurdles ahead to bring a proposed GapmeR technology to clinics, these can be overcome by engaging collaborative academia-industry partnerships, setting-up joint screening, and testing facilities and making research funding available to support new concepts. We believe that targeted GapmeR in combination with recombinant ACE2 protein could provide a viable rapidly implementable therapeutic approach for COVID-19 and similar infections in the future.

## Data Availability Statement

Publicly available datasets were analyzed in this study. This data can be found here: https://www.ncbi.nlm.nih.gov/labs/virus/vssi/#/virus?SeqType_s=Nucleotide&VirusLineage_ss=SARS-CoV-2,%20taxid:2697049&utm_source=gene&utm_medium=referral&utm_campaign=COVID-19.

## Author Contributions

NV and DK conceived the idea. NV, MF, SD, and DK wrote the manuscript. All authors approved the final manuscript version.

## Conflict of Interest

The authors declare that the research was conducted in the absence of any commercial or financial relationships that could be construed as a potential conflict of interest.

## Footnotes

1 https://covid19.who.int

2 https://clinicaltrials.gov
